# Non-diagnostic autopsy findings in sudden unexplained death victims

**DOI:** 10.1186/s12872-020-01361-z

**Published:** 2020-02-04

**Authors:** Puriya Daniel Yazdanfard, Alex Hørby Christensen, Jacob Tfelt-Hansen, Henning Bundgaard, Bo Gregers Winkel

**Affiliations:** 1 Department of Cardiology The Heart Center, Copenhagen University Hospital Rigshospitalet, Blegdamsvej 9, 2142, 2100 Copenhagen, Denmark; 2 Department of Cardiology, Herlev-Gentofte Hospital, Copenhagen University Hospital, Copenhagen, Denmark; 3 Department of Forensic Medicine, Faculty of Medical Sciences, University of Copenhagen, Copenhagen, Denmark

**Keywords:** Autopsy, Autopsy findings, Cardiac disease, Non-diagnostic findings, Sudden Cardiac Death, Sudden Unexplained Death, Sudden Arrhythmic Death Syndrome, SCD, SUD, SADS

## Abstract

**Background:**

Several inherited cardiac diseases may lead to sudden cardiac death (SCD) a devastating event in the families. It is crucial to establish a post mortem diagnosis to facilitate relevant work-up and treatment of family members. Sudden unexplained death (SUD) victims constitute roughly one third of all SCD cases in Denmark.

**Methods:**

This was a single center, retrospective study investigating SUD cases. Victims who died unexplained due to suspected or confirmed cardiac disease were consecutively referred to a third line referral center established in 2005. All autopsy reports were investigated. Victims were divided into two groups: non-diagnostic cardiac findings and normal cardiac findings. None of the included victims had findings consistent with a diagnosis based on existing criteria.

**Results:**

In total, 99 SUD cases were referred. The mean age of the victims was 37 years (range 0–62 years, 75% males). A total of 14 (14%) victims had a cardiovascular diagnosis pre-mortem. Thirty-seven cases had normal cardiac findings and non-diagnostic cardiac findings were found in 62 cases (63%). The five most common findings included ventricular hypertrophy and/or enlarged heart (*n* = 35, 35%), coronary atheromatosis (*n* = 31, 31%), myocardial fibrosis (*n* = 19, 19%), dilated chambers (*n* = 7, 7%) and myocardial inflammation (*n* = 5, 5%).

**Conclusion:**

One third of SUD victims had normal cardiac findings and non-diagnostic cardiac findings were seen in almost two thirds of the SUD victims. These non-diagnostic findings may be precursors or early markers for underlying structural cardiac disorders or may be innocent bystanders in some cases. Further studies and improved post-mortem examination methods are needed for optimization of diagnostics in SUD.

## Background

Sudden cardiac death (SCD) leads to tragedies in families with huge social and psychological consequences and is an important contributor to years of life lost among the young. It is crucial to establish a post mortem diagnosis to facilitate relevant treatment of family members and avoid further deaths as inherited cardiac diseases play an important role in SCD of the young.

SCD in the young is attributed to a variety of causes of which more than a third is due to ischemic heart disease [1]. Other reasons include cardiomyopathies (hypertrophic, dilated and arrhythmogenic cardiomyopathy), myocarditis, aortic disease, valvular heart disease, and congenital heart defects. In cases where autopsies have not reported structural cardiac abnormalities, death may be due to an underlying primary arrhythmogenic disease such as long or short QT syndrome (LQTS/SQTS), Brugada Syndrome (BrS), and catecholaminergic polymorphic ventricular tachycardia (CPVT) [2, 3]. Establishing a post mortem diagnosis remains challenging despite standardized autopsy guidelines [4] and review of known medical conditions. In a nationwide setting, up to 31% of SCD victims below age 50 years remain undiagnosed after autopsy [1]. However, even though the autopsy does not provide a cause of death, unspecific cardiac findings with questionable causality to the occurred death are often reported. Very few systematic presentations of these autopsy findings have been reported before [5].

The purpose of this study was to systematically present non-diagnostic autopsy findings in sudden unexplained death (SUD) victims.

## Methods

### Study design and population

This study was a single center, retrospective study investigating SUD cases. Cases with patients who died suddenly, unexpected and unexplained due to suspected or confirmed cardiac disease were consecutively referred to our third line referral center, Rigshospitalet, Copenhagen University Hospital, Denmark for evaluation. The institution covers the entire Zealand region of Denmark (total population 2.66 mill). For the present analysis, we included all referred cases that had not received a conclusive post mortem diagnosis after careful review of all available data, including autopsy reports and prior medical records. Victims for whom toxicology results indicated a likely intoxication as the cause of death were also excluded from the analysis. The autopsied victims were classified into two different groups: victims with non-diagnostic cardiac findings and victims with a normal autopsy (no findings).

All medical records and autopsies reports were acquired digitally. All individuals in Denmark have a unique civil registration number that can be used to acquire archived information. The electronic records contain information from multiple sources including data on medical history, received treatments and paraclinical findings, including autopsy reports. The records were meticulously investigated for all relevant information on the autopsied victims.

### Autopsies

In Denmark, forensic autopsies are requested when the cause of death cannot be established by a full exterior examination of the corpse or in relation to police investigations. Coroners from Departments of Forensic Medicine do a complete autopsy of all organs following a standardized protocol. Autopsies are always supervised by another forensic pathologist. All findings are presented and findings of significance are given in a conclusion. Histopathology is conducted routinely and if deemed relevant toxicology screening is performed as well. Hospital autopsies are conducted in the local hospital’s pathology department. These can be requested by a physician with consent from relatives when no forensic autopsy has been ordered.

### Definitions

SCD were defined in autopsied cases as the sudden, natural and unexpected death of unknown or cardiac cause. In unwitnessed cases the deceased person had to be seen alive and functioning normally no more than 24 h before being found dead, and in witnessed cases an acute change in cardiovascular status had to have occurred with the time to death being less than one hour [6, 7]. Unexplained SCD cases were categorized as sudden unexplained death (SUD). In addition, sudden arrhythmic death syndrome (SADS) was a subdivision of SUD requiring a negative toxicology alongside the negative autopsy.

Autopsies were considered normal or blank (no extra-cardiac or cardiac related findings) if all measures were within normal range as defined [8]. Non-diagnostic findings were those that did not fulfill criteria for neither a structural normal heart, nor a specific disease [1, 8].

Microscopic and macroscopic criteria for non-diagnostic cardiac abnormalities were as follows: Hypertrophic and/or enlarged Heart: Unexplained localized or concentric hypertrophy (> 15 mm) of the left ventricular wall and/or abnormal heart weight when corrected for body surface area (> 0.5% of total body weight or above 500 g), not fulfilling criteria for hypertrophic cardiomyopathy (no myocyte disarray). Coronary artery atheromatosis: The presence of coronary artery atherosclerosis being less than 75% of the circumference of the arterial vessel, with no signs of stenosis or acute myocardial infarction. Myocardial fibrosis: Any degree of myocardial fibrosis in the left and/or right ventricle without any concomitant signs of structural or ischemic heart disease. The location of fibrosis can be focal (gathered in a region, eg. septum), Diffuse (evenly spread over the entire myocardium) and patchy (being gathered in patches over multiple myocardial regions). Dilated chambers: Dilatation of the left/right ventricle (measured transverse and longitudinal intraventricular size [4]) with or without wall thinning but with no evidence of significant fibrosis. Congenital heart defects: Presence of congenital defects, including but not limited to patent foramen ovale (in victims older than 5 years of age) and septal defects, but not considered a likely substrate for ventricular arrhythmias or heart failure-related death. Myocardial inflammation: Low degree of leukocyte or neutrophilic infiltrations of the myocardium with or without fibrosis. Not considered pathognomic for myocarditis, due to not having any presence of necrosis within the examined area, thus not fulfilling revised Dallas criteria [9]. Valvular heart disease: Presence of valvular heart disease, but not of a severity to be the likely cause of death.

Additionally, non-diagnostic findings unrelated to cardiac abnormalities were defined as follows: Atherosclerosis of the great vessels: Any degree of atherosclerosis described by the coroner in the arterial vessels of the body. Aspiration: The presence of ventricular substance within the airways. Cerebral hemorrhage: The presence of hemorrhage within the cranial cavity not considered as cause of death. Pneumonia: The presence of lymphocyte infiltrations within the lungs, not considered trivial but likewise not considered as the cause of death.

### Statistical analysis

Data is presented as number (percentage), mean ± standard deviations (SD). Statistical analysis was performed using two sampled means comparisons tests (t-test). Where appropriate linear regressions were performed with Pearson’s correlation. *P* value of less than 5% were considered significant. Confidence intervals were set at 95%. Data were processed using STATA 13.0 (StataCorp, USA).

## Results

### Study population

In total, 99 SUD cases were identified. The age of the victims was between 0 and 62 years (mean 34.3 years, SD 14.4) and 74 were men (75%). Two victims were under 1 year of age (Table 1). Twenty-nine (29%) of all SUD victims could be classified as SADS as they had a negative toxicology screen performed. SADS victims were younger than SUD victims (mean age 28 years compared to 37 years, *p* = 0.048).

**Table 1:** Characteristics of the Sudden Unexplained Death (SUD) population (*n* = 99)

Age at death, years	34.3 (range 0–62)
Male sex	74 (75%)
Prior cardiovascular work-up	21 (21%)
Preexisting cardiovascular diagnosis	14 (14%)
Preexisting psychiatric diagnosis	8 (8%)

### Previous health status

Among all victims 21 (21%) had a cardiac assessment performed prior to their death. All had electrocardiograms taken (*n* = 21, 100%), 11 (52%) had echocardiography, five (23%) had cardiac CT or MRI, four (19%) had coronary angiographies, four (19%) had Holter monitorings, and four (19%) had an exercise test performed. One (5%) victim was previously cardioverted for atrial fibrillation.

Fourteen (14%) had at least one established cardiovascular diagnosis during their lifetime: hypertension (*n* = 5, 36%), atrial fibrillation/flutter (*n* = 4, 29%), dyslipidemia (n = 4, 29%), valvular heart disease (*n* = 3, 21%), second degree AV block (*n* = 1, 7%), acute myocardial infarction (n = 1, 7%), and stroke (n = 1, 7%). In addition, 8 victims (8%) had a psychiatric diagnosis (schizophrenia/bipolar disorder/depression, all treated with QT prolonging drugs).

We observed no difference in having had a cardiac assessment prior to death compared to being in the group with non-diagnostic autopsy findings or having a blank autopsy (*p* = 0.79). Nor did we observe any difference in victims having had a cardiac related diagnosis prior to death and being in the group with non-diagnostic autopsy findings or having a blank autopsy (*p* = 0.60).

### Autopsy findings

**Fig. 1:**
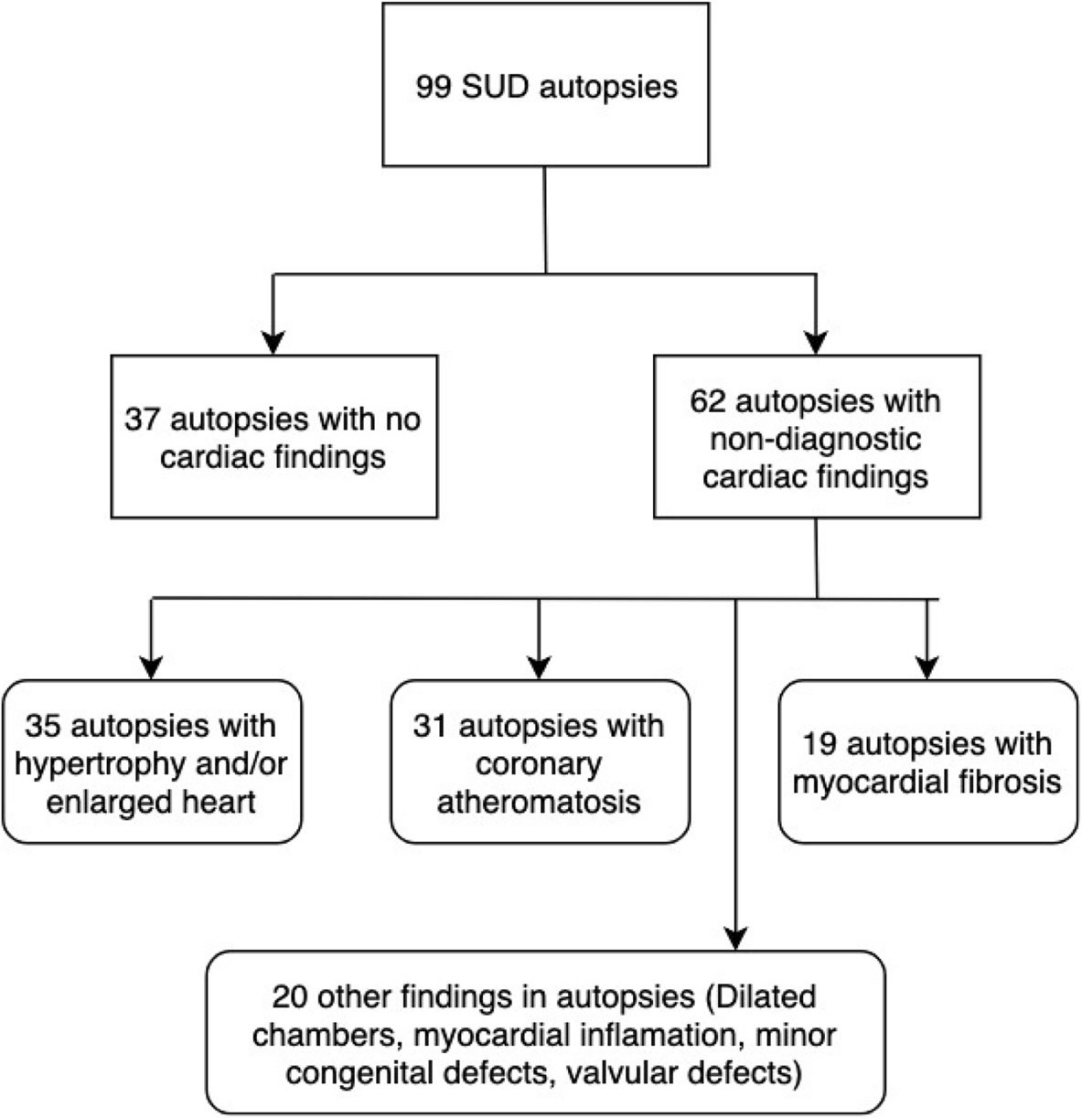
Victims of Unexplained Sudden Cardiac Death. Flowchart with included SUD victims. SUD = Sudden Unexplained Death

Non-diagnostic cardiac findings were reported in 62 cases (63%) and normal cardiac findings were found in the remaining 37 individuals (37%) (Fig. 1). SADS and SUD victims had the same amount of non-diagnostic findings (*p* = 0.64). Non-diagnostic autopsy findings are presented in Table 2. The three main findings in the 62 cases with non-diagnostic findings included ventricular hypertrophy and/or enlarged heart (*n* = 35, 56%), coronary atheromatosis (*n* = 31, 50%) and myocardial fibrosis (*n* = 19, 31%). In 10 victims, fibrosis was described being diffuse/spread interstitial. Five victims had focal spots of fibrosis (three of these in the posterior left ventricular wall, one subendocardial fibrosis, one had septal fibrosis) and finally one victim had fibrosis of the conduction system. In three cases specific information regarding fibrosis was not available. The co-presence of fibrosis and hypertrophy/enlarged heart was seen in 12 (19%) victims.

**Table 2:** Autopsy Findings from 99 Victims of Sudden Unexplained Death (SUD)

Cardiac findings	62 (63%)
Blank Autopsy	37 (37%)
Heart weight, grams	416^*^ (240–660)
LV thickness, mm	14.7^*^ (5–25)
RV thickness, mm	3.9^X^ (0.3–15)
Mean BMI, kg/m^2^	24.6^†^ (15–37)
Heart weight / Body Mass, ratio	0.5%^†^ (0.3–0.8)
Hypertrophy and/or enlarged heart	35 (35%)
Coronary atheromatosis	31 (31%)
Myocardial fibrosis	19 (19%)
Dilated chambers	7 (7%)
Myocardial Inflamation	5 (5%)
Minor congenital heart defects	5 (5%)
Valvular heart defects	3 (3%)
Non-cardiac findings	26 (26%)
Atherosclerosis of the great vessels	24 (24%)
Aspiration	2 (2%)
Cerebral haemorrhage	1 (1%)
Pneumonia	1 (1%)
Toxicology performed	29 (29%)

Abbreviations: *BMI* body mass index; *LV* left ventricle; *RV* right ventricle.^†^*n* = 47; ^x^n = 60; *n = 61

None of the findings fulfilled diagnostic criteria. All percentages are given out of total the total population

Cardiac mass was significantly larger for men compared to women after correction for body surface area (BSA); 217 g/m^2^ vs 173 g/m^2^, *p* = 0.0026). We observed a relationship between age and BSA corrected cardiac mass (r = 0.57, *p* < 0.001).

Dilated chambers were present in 7 (11%) cases with non-diagnostic findings. Three of these victims had both dilated left and right ventricle with normal microscopic findings. Two victims had dilated left ventricles with microscopic evidence of fat infiltrations in the right ventricle and one victim had biventricular dilatation and microscopic evidence of fat infiltrations in the right ventricle. However, none of these victims fulfilled the criteria for having arrhythmogenic right ventricular cardiomyopathy. The last victim had a dilated left ventricle and moderate ventricular hypertrophy.

Minor congenital defects were seen in 5 victims with non-diagnostic findings (8%) and included three victims with patent foramen ovale (aged 18, 28, 42 years), one victim had an atrial septal defect (age 17 years), finally in one victim no specific information was available other than it was ‘minor’ (age 32 years). Myocardial inflammation was seen in 5 (8%) victims with non-diagnostic findings: one victim with minimal signs of inflammation, one victim with acute inflammatory signs (predominantly neutrophilic cells), one victim with inflammation and eosinophilia possibly due to hypersensibility, one victim with chronic inflammatory cells in the myocardium, and finally no specific information was available on the last victim. Valvular heart defects were seen in 3 (5%) victims with non-diagnostic findings and included one victim with dilated mitral and tricuspidal valves with universally dilated chambers. Two victims had moderate degrees of mitral valve calcifications, but no significant stenosis.

## Discussion

Non-diagnostic findings were identified in 62 (63%) of SUD cases. The most common findings were hypertrophy/enlarged heart in 35 (35%), coronary artery atherosclerosis in 31 (31%), and myocardial fibrosis in 19 (19%). In total 21 (21%) had a cardiovascular evaluation prior to death, with 14 (14%) having been diagnosed with a cardiovascular disorder, mainly hypertension (*n* = 5), atrial fibrillation/flutter (*n* = 4), and dyslipidemia (*n* = 4).

Our findings are in agreement with a recent study of 98 SUD cases [5], in which 60% of the cases were categorized with non-diagnostic findings, mainly related to presence of left ventricular hypertrophy, cardiomegaly, inflammation and fibrosis.

While not diagnostically conclusive it is well established that left ventricular hypertrophy increases risk of mortality independently of other factors [10]. In extension to this a conceivable increase in risk of ventricular arrhythmias has been reported in patients with left ventricular hypertrophy [11, 12]. Experiments in animal models have shown left ventricular hypertrophy increases refractoriness and prolongation of action potentials leading to increased vulnerability to arrhythmias [13]. In addition, increased cardiac mass leads to rising pressure on vessels which ultimately reduces perfusion of the myocardium increasing susceptibility to ischemic damage and scarring [13].

As depicted in Tables 2, 19 (19%) of the victims had some degree of cardiac interstitial fibrosis. The degree of fibrosis was not sufficient to reach a diagnostic conclusion, however the literature suggests an association between non-specific interstitial fibrosis and ventricular arrhythmias due to reentrant mechanisms [2]. These reentries can occur due to several reasons, whether it be resulting in slow and fast pathways that can facilitate a reentry circuit or reentries around a focal scar than can operate as an ectopic point sending out electrical impulses to the rest of the heart [14, 15]. Fibrosis may represent cellular degeneration due to hypoxia or spontaneous cellular degeneration due to an underlying genetic cause such as hypertrophic cardiomyopathy (HCM). Several studies have shown the presence of fibrosis in the myocardium and the activation of profibrotic pathways can be an early predictor for HCM despite the lack of ventricular hypertrophy [16, 17]. While the literature does exclude the possibility that the victims had early manifestations of HCM little evidence exists that link these unspecific cardiac alterations to SCD. While the unspecific findings may be a precursor for disease, it could also merely be age-related cardiac phenonenoms [18].

One of the hallmarks of hypertrophic cardiomyopathy is the presence of myocardial disarray in conjunction with hypertrophy and fibrosis of the heart [19]. We found 12 (12%) victims with non-diagnostic fibrosis and/or hypertrophy/enlargement of the heart. None of these victims had histologically verified myocyte disarray, yet it is possible the disarray is focal and patchy not involving the entire myocardium [20]. One study based on late gadolinium enhancement cardiac MR imaging concludes that patients with identifiable HCM mutations more often had localized focal cardiac fibrosis instead of diffuse cardiac fibrosis [21]. Thus, the selected sites for microscopic investigation may have missed the disarray. On the contrary, a hypertrophic heart could also point towards hypertension as the primary cause, therefore having little to no relevance in the cause of death. Although not formally quantified the typical description of the fibroses was diffuse, which may indicate that the victims had hypertension and other non-HCM causes [22–24]. Sudden death is unlikely to be the first finding as the result of hypertension, still it has been seen that hypertension and left ventricular hypertrophy can result in premature ventricular activity that in rare cases can lead to fatal arrhythmias [25]. However, we cannot rule out that the hypertrophic/enlarged heart could also be an innocent bystander not related to the victim’s death.

Dilated heart chambers were seen in 7 (7%) of the victims. It is possible this finding may represent a variation of dilated cardiomyopathy (DCM). In a Danish nationwide study 3% of the autopsied-explained SCD victims had DCM [1]. The clinical manifestations of DCM are heterogeneous, but most cases present with heart failure symptoms but the presentation can be SCD [26]. Post mortem diagnosis of DCM is performed by observing the gross heart size/weight and dilated ventricles and may be supported by genetic testing. Roughly 30–50% of DCM cases are confirmed via genetics while other causes can be due to autoimmunity, narcotics and viral infections [26]. Viral infections have been linked to heart failure and DCM [27–30]. While the mechanism still remains uncertain some believe that a combination of stress-induced cellular apoptosis due to high viral RNA load and T-cell mediated cell death are important factors in the development of DCM [27]. Low degrees myocardial inflammation was seen in 5 victims, but the degree of lymphocyte infiltration was deemed unlikely to be the cause of death. However, only one victim had both dilated chambers and myocardial inflammation. Inflammatory findings are also typical findings post myocardial infarction and one study described co-occurrence of myocardial infarction and myocarditis [31]. Based on existing knowledge myocardial inflammation can result in multiple outcomes, however it may also be a non-relevant coincidental finding within these victims.

Thirty-one (31%) of the victims with non-diagnostic findings had some degree of coronary artery atheromatosis. In these cases, it is exceedingly difficult to assess the importance of the atherosclerosis in the coronary arteries since often it could be a coincidental finding. One study reported that atherosclerosis in the coronary vessels was present in 73% of a group of deceased individuals that died from non-cardiac causes [32]. However, it is also well known that thrombus autolysis can occur spontaneously, and it takes several hours for the myocardial ischemic changes to be microscopically visible. Therefore, we cannot exclude that some victims may have died due to myocardial infarction, but post mortem investigations were without diagnostic findings disclosing rapid thrombus autolysis. Coronary artery atheromatosis and atherosclerosis of great arteries are often seen together. We reported a total rate of 24% atherosclerosis in the major arteries in the victims with non-diagnostic findings. It is well recognized that light degrees of atherosclerosis is seen in healthy individuals progressing as a person ages [33].

In total 37 (37%) of all victims had no cardiac findings on autopsy. In cases with lack of structural cardiac abnormalities deaths may be attributed to inherited arrhythmogenic disorders (i.e. BrS, LQTS, SQTS, CPVT) [34, 35]. These channelopathies are diagnosed by ECG, exercise ECG, and ECG-induced drug-challenges making a post mortem diagnosis impossible. The autopsy usually presents without structural cardiac findings. Although purely speculative, subtle findings in otherwise autopsy-negative cases of SCD might also be due to primary arrhythmogenic disorders. For instance, it is well known that variants in the gene *SCN5A* phenotypically can give rise to both structural cardiac disease (DCM), conduction abnormalities, and primary arrhythmogenic disorders (LQTS and BrS) [36]. In these cases the post mortem diagnosis relies on genetic testing with many genetic variants remaining unclassifiable and variable penetrance/expressivity hampering cosegregation analyses in families [37–41]. At our institution we do not routinely perform genetic testing in unexplained SCD cases. While a molecular autopsy could be used in autopsied cases which fulfills diagnostic criteria for certain cardiomyopathies (e.g. HCM, DCM, and ARVC). Variants of unknown significance (VUS) as well as the lack of genotype-phenotype correlation can make interpretation difficult, especially in unexplained SCD cases. Current guidelines state that targeted post-mortem genetic analysis of potentially disease-causing genes should be considered in all sudden death victims in whom a specific inheritable channelopathy or cardiomyopathy is suspected (class IIa, level of evidence C) [42].

### Limitation

The retrospective nature of the study unfortunately led to unavoidable missing data points. We did not compare the amount of cardiac non-diagnostic autopsy findings in our cohort with a control group, this was however done in a recent study were they found far more non-diagnostic findings in the SUD cohort compared to cardiac healthy controls [5]. In relations, it is also a limitation that we did not have access to family evaluation in this study. Moreover, we have not performed systematic molecular autopsies. Molecular autopsy might have resulted in diagnosing some SCD cases. In addition, our center is a referral center, thus victims are referred to this center for evaluation, this may ultimately result in selection bias. Furthermore, autopsy protocols evolved over time resulting in variations among autopsies.

## Conclusion

In a referred cohort of SUD cases, unspecific cardiac findings were seen in 63% of the autopsied with the most common findings including hypertrophy/enlargement of heart, coronary artery atheromatosis and diffuse fibrosis. These unspecific findings may be precursors or early signs of underlying structural cardiac disorders but could also be spur findings in patients with inherited arrhythmogenic disorders. In total, 37% of all victims had no cardiac finding on autopsy. These cases could represent underlying inherited arrhythmogenic disorders, as has been shown in previous studies.

## Data Availability

The datasets used and/or analyzed during the current study are available from the corresponding author on reasonable request.

## Abbreviations

ARVC: Arrhythmogenic right ventricular cardiomyopathy
BrS: Brugada syndrome
BSA: Body surface area
CPVT: Catecholaminergic polymorphic ventricular tachycardia
DCM: Dilated cardiomyopathy
HCM: Hypertrophic cardiomyopathy
LQTS: Long QT syndrome
SADS: Sudden arrhythmic death syndrome
SCD: Sudden cardiac death
SD: Standard deviations
SQTS: Short QT syndrome
SUD: Sudden unexplained death
VUS: Variants of unknown significance
